# Phytochemical Characterization and In Vitro Biological Activities of *Macleania rupestris* (Ericaceae): Insights into Nutraceutical Potential

**DOI:** 10.3390/molecules30214251

**Published:** 2025-10-31

**Authors:** Arianna Mayorga-Ramos, Rebeca Gonzalez-Pastor, Juan A. Puente-Pineda, Carlos Barba-Ostria, Eduardo Tejera, Diana Celi, Patricio Rojas-Silva, Ana Belén Peñaherrera-Pazmiño, Linda P. Guamán

**Affiliations:** 1 Centro de Investigación Biomédica, Facultad de Ciencias de la Salud Eugenio Espejo, Universidad UTE, Quito 170129, Ecuador; arianna.mayorga@ute.edu.ec (A.M.-R.); rebeca.gonzalez@ute.edu.ec (R.G.-P.); juan.puente@ute.edu.ec (J.A.P.-P.); ana.penaherrera@ute.edu.ec (A.B.P.-P.); 2 Escuela de Medicina, Colegio de Ciencias de la Salud, Universidad San Francisco de Quito, Quito 170901, Ecuador; 3 Instituto de Microbiología, Colegio de Ciencias Biológicas y Ambientales COCIBA, Universidad San Francisco de Quito, Quito 170901, Ecuador; projas1@usfq.edu.ec; 4 Bio-Cheminformatics Research Group, Universidad de Las Américas, Quito 170504, Ecuador; eduardo.tejera@udla.edu.ec (E.T.); diana.celi@udla.edu.ec (D.C.); 5 Facultad de Ingeniería y Ciencias Aplicadas, Carrera de Biotecnología, Universidad de Las Américas, Quito 170504, Ecuador

**Keywords:** *Macleania rupestris*, anthocyanins, phytochemical profiling, hemolytic activity, anti-tumoral activity, anti-inflammatory activity

## Abstract

The Ericaceae family encompasses several berries with recognized health-promoting properties; however, *Macleania rupestris*, a neotropical species endemic to the Andean region, remains poorly characterized. Background/Objectives: This study aimed to identify the chemical composition of *M. rupestris* ethanolic extracts and evaluate their biological activities, including antitumoral, hemolytic, anti-inflammatory, and leishmanicidal effects. Methods: The *M. rupestris* ethanolic extracts were obtained from lyophilized fruits and analyzed by HPLC-MS/MS for phytochemical profiling. Bioactivities were assessed in vitro using tumor and non-tumor cell lines (MTT assay), erythrocyte hemolysis assays, RAW 264.7 macrophage inflammation models, and *Leishmania mexicana* promastigotes. Results: The chemical analysis revealed anthocyanins (cyanidin-3-glucoside, malvidin-3-glucoside, petunidin-3-glucoside, delphinidin-3-arabinoside), flavonols (quercetin and myricetin derivatives), and coumaroyl iridoids. The extract showed modest antiproliferative activity (IC_50_ 10.4–22.5 mg/mL) across tumor cell lines with low therapeutic indices, indicating limited selectivity. In contrast, hemolytic activity was negligible (<5% at all tested concentrations), suggesting high biocompatibility. Anti-inflammatory assays indicated a dose-dependent reduction in nitric oxide (NO) production, while no significant leishmanicidal activity was detected. Conclusions: This study provides the first comprehensive evaluation of the previously listed *M. rupestris* bioactivities. While its antitumoral effects appear limited, its strong hemocompatibility and presence of antioxidant metabolites highlight its potential for biomedical and nutraceutical applications where biocompatibility is critical. Further studies are needed to optimize bioactivity and explore potential synergistic effects.

## 1. Introduction

Berries of the family *Ericaceae* are well recognized as reservoirs of bioactive compounds, particularly anthocyanins, flavonoids, and iridoids, which contribute to their antioxidant, anti-inflammatory, and potential anticancer properties [1]. Numerous studies have demonstrated that consumption of blueberries, cranberries, and related species may reduce the risk of chronic diseases, including cardiovascular disorders and certain cancers, largely due to their polyphenolic content and capacity to modulate oxidative stress and cellular signaling pathways [2,3]. Despite this growing body of evidence, several neotropical species remain underexplored, limiting our understanding of their phytochemical diversity and therapeutic potential.

*Macleania rupestris* (Kunth) A.C.Sm., an endemic berry of the Ecuadorian Andes, belongs to this under-represented group. Traditionally consumed in local communities, its chemical composition and biological properties have not been systematically characterized, although related genera within the *Ericaceae* have been linked to significant health-promoting effects [4,5,6]. Given the ecological uniqueness of the Andean region and the selective pressures that shape secondary metabolite biosynthesis, *M. rupestris* represents a promising candidate for bioprospecting novel phytochemicals with pharmacological applications.

Across *Ericaceae*, berry extracts share a canonical phytochemical core enriched in anthocyanidin 3-O-glycosides (e.g., cyanidin/delphinidin/malvidin) and flavonol O-glycosides (quercetin/myricetin), which underlie widely reported antioxidant and anti-inflammatory effects [7]. *M. rupestris*, while consistent with this core, has been suggested to display a broadened metabolite space that includes acylated iridoid-type constituents alongside polyhydroxylated flavonoids—traits that point to lineage-specific biosynthetic features within *Ericaceae*.

This study sought to characterize the chemical profiles of *M. rupestris* extracts and evaluate their biological activities across multiple models. Here, we profile its chemical composition and evaluate biocompatibility- and inflammation-related activities to position *M. rupestris* within the *Ericaceae* landscape. Specifically, we investigated cytotoxicity in tumor and non-tumor cell lines, hemolytic activity, anti-inflammatory properties, and leishmanicidal potential. By integrating phytochemical identification through HPLC-MS/MS with in vitro assays, our work provides the first comprehensive assessment of *M. rupestris* as a source of bioactive compounds. The results expand the current knowledge on Andean berries and contribute to the broader understanding of *Ericaceae* as valuable reservoirs for biomedical and nutraceutical applications.

## 2. Results

### 2.1. Chemical Characterization

**Figure 1:**
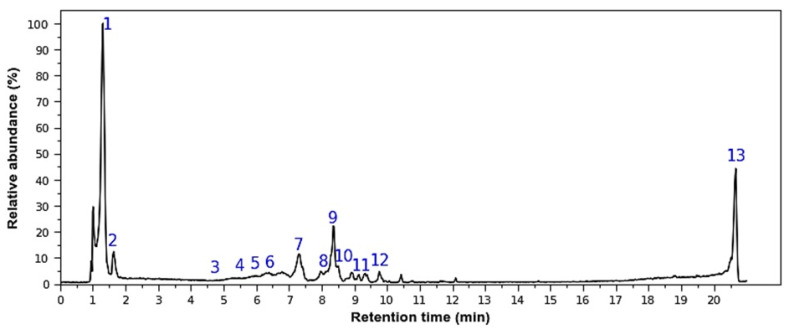
Representative HPLC-DAD Chromatogram of *M. rupestris* extract. Peak numbers (1–13) in the chromatogram correspond to the metabolite IDs (ID 1–13) tentatively identified in Table 1.

The chemical composition of *M. rupestris* was first analyzed by a High-Performance Liquid Chromatography–Diode Array Detector (HPLC-DAD) Chromatogram (Figure 1) and later by High-Performance Liquid Chromatography–Tandem Mass Spectrometry (HPLC-MS/MS); the tentative identification of the parent ions is presented in Table 1 in positive and negative ions modes. Some of the molecules are only presented in one mode because no ion was detected in the other ionization mode.

The ID1 peak at *m*/*z* 381 was previously identified by [8] as sucrose, a disaccharide with the adduct [M + K]^+^. The mass pattern includes a dominant ion at 219 *m*/*z* (with adduct [M + K]^+^) and a less intense peak at 201 *m*/*z* [M + K − H_2_O]^+^. Both ions correspond to monosaccharides (hexoses) derived from the parent sucrose molecule. In ID2 the rupture of 325 *m*/*z* is matched in MzCloud (82.4%) as a fragment (dimer) of raffinose. However, raffinose has a molecular mass of 504, indicating that while the deprotonated hexose-pentose dimer (of raffinose) is probably a tentative annotation, the raffinose is not. The loss of 78 uma from 403 → 325 ([M + H − C_2_H_6_O_3_]^+^) could suggest a 1–2 or 1–3 disaccharide linkage [9]. Therefore, *m*/*z* 325 is probably some sort of disaccharide, while 403 is probably an indication of a modification of any of the sugar units.

Four anthocyanins were identified, all with an adduct of type [M]^+^. The anthocyanins with parent masses of 435 *m*/*z* (ID3) and 449 *m*/*z* (ID4), eluted at retention times (RT) of 4.75 and 5.98 min, respectively, and were previously identified as delphinidin-3-arabinoside and cyanidin-3-glucoside, as reported by [10]. The fragment at 303 *m*/*z*, which is consistent with delphinidin, was accompanied by a neutral loss of 132 Da, indicating the presence of a pentoside moiety. The fragmentations at 287 *m*/*z* and 317 *m*/*z* confirmed the identity of ID4 as cyanidin-3-glucoside. This identification was further validated using an internal standard. The other two anthocyanins (ID5 and ID6), with molecular ions of 479 *m*/*z* (RT = 5.47 min) and 493 *m*/*z* (RT = 6.40 min), were previously identified as petunidin-3-glucoside and malvidin-3-glucoside by [11]. The 479 *m*/*z* ion showed a fragment at 317 *m*/*z*, corresponding to petunidin, with a neutral loss of 162 Da, indicating the presence of a glucoside. For the 493 *m*/*z* ion, the main fragment at 331 *m*/*z* ([M-162]^+^) confirmed the presence of malvidin-3-glucoside, reflecting a neutral loss of 162 Da [12].

The rupture of 319 *m*/*z* (ID7) is consistent with myricetin (94.9% score). The loss of 162 uma is consistent with the hexose moiety. The MzCloud proposes the myricetin 3-o-galcotpyranoside, which is consistent with the identified pattern. However, in general terms should be a myricetin-3-o-hexoside. The precursor ion at *m*/*z* 519 (retention time = 8.36 min) (ID 9) was identified in MzMine as vaccinoside, a type of coumaroyl iridoid (score 79%). However, the molecular mass of vaccinoside is 536 Da, which differs from the value previously reported. One potential explanation is that the detected ion is an adduct, specifically [M + H − H_2_O]^+^, as previously reported in the study of several *Macleania* species [12]. A comparable fragmentation pattern has also been documented in the case of andromedoside. To confirm the identity of the ion, a fragmentation spectrum was obtained in negative ionization mode, which detected a parent ion at *m*/*z* 535, consistent with the molecular mass of vaccinoside. The resulting fragmentation pattern is as follows: 535→371 (100), 191 (95), 448 (80), 241 (60), 373 (40), 491 (20), 163 (15) (Table 1). This is consistent with the fragmentation of several coumaroyl iridoid isomers [13,14]. Nevertheless, the precise isomer remains unidentified in this instance. Consequently, we have designated it as ‘a type of coumaroyl iridoid’ in ID 9.

IDs 8 and 11 correspond to the masses 465 *m*/*z* and 435 *m*/*z*, respectively, both of which exhibit a prominent peak at *m*/*z* 303. Based on its mass, the fragment at *m*/*z* 303 was identified as the quercetin aglycone ion, the fragmentation of which was confirmed with an internal standard. The mass 465 *m*/*z* has an MzCloud match score of 85% and has been tentatively assigned to quercetin 3-galactoside, based on its fragmentation pattern. The difference in mass from 435 to 303 *m*/*z* (132 Da) may correspond to the loss of a pentoside. In this case, MzCloud proposes avicularin (score 92.9%). However, we prefer to use the general terms quercetin hexoside and quercetin pentoside, respectively.

The mass fragmentation pattern of ID 10 at *m*/*z* 495 was identified as methoxy-myricetin in the GNPS database, with a score of 83%. The mass difference between 495 and 333 *m*/*z* (162 Da) is attributed to the neutral loss of a hexoside. Nevertheless, hydroxyflavans (e.g., nanupetin) and monomethoxyflavones (e.g., naricitrin) also exhibit a molecular ion at *m*/*z* 333. However, the 179 *m*/*z* in negative ionization could suggest a retro-diels-alder fragmentation [15] of myricetin, supporting the possible annotation of this particular flavonoid. ID12 exhibits a molecular ion peak at 509 *m*/*z* with a fragment at 347 *m*/*z* ([M − 162 + H]^+^), indicative of the loss of a hexoside. This pattern has previously been identified as syringetin-3-O-hexoside in various *Macleania* species [12]. Furthermore, the remaining fragment at 491 *m*/*z* corresponds to a neutral loss of 18 Da, which is consistent with the loss of water, and the negative rupture is almost identical to the spectra shown in the PubChem database for syringetin-3-o-glucoside. The peak characterized by the molecular ion 288 *m*/*z* (ID13) was not identified and labeled as UI.

**Table 1:** Tentative metabolites identified in *M. rupestris* by HPLC-ESI-MS/MS in positive and negative ionization mode.

ID	Positive/ Negative	RT	*m*/*z*	MS/MS	Tentative Identification	Reference
1	P	1.38	381	219 (100), 201 (35), 363 (15)	Disaccharide (putative)	[8]
2	P	1.41	403	325 (100), 289 (15), 271 (5) 325→289 (100), 271 (30), 307 (25), 163 (20)	Disaccharide-like ion (putative)	MzCloud 82.4%
3	P	4.75	435	303 (100)	Delphinidin-3-arabinoside	[10]
4	P	5.47	479	317 (100)	Petunidin-3-glucoside	[11,16]
5	P	5.98	449	287 (100), 317 (46)	Cyanidin 3-glucoside	(**)
6	P	6.40	493	331 (100)	Malvidin-3-glucoside	[11,16]
7	P	7.31	481	319 (100) 319→273 (100), 301 (45), 165 (35), 245 (30), 153 (30)	Myricetin 3-o-hexoside	MzCloud 94.9%
	N		479	317 (100), 271 (10)		
8	P	8.29	465	303 (100), 319 (20), 333 (15) 303→257 (100), 285 (70), 229 (65), 165 (65), 247 (35)	Quercetin-hexoside	(**)
	N		463	301 (100), 151 (10)		
9	P	8.36	519	339 (100), 357 (90), 175 (50), 193 (25), 309 (20), 165 (10) 357→147 (100), 193 (30), 165 (10)	Acylated iridoid glycoside (vaccinoside) (putative)	
	N		535	371 (100), 191 (95), 448 (80), 241 (60), 373 (40), 491 (20), 163 (15)		
10	P	8.50	495	333 (100), 477 (5)	O-methyl-myricetin O-hexoside	GNPS 83%
	N		493	331 (100), 330 (45), 315 (15), 413 (8), 179 (5)		
11	P	8.92	435	303 (100), 361 (6) 303→257 (100), 285 (70), 229 (65), 165 (65), 247 (35)	Quercetin O-pentoside	MzCloud 92.9%
	N		433	301 (100)		
12	P	9.76	509	347 (100), 491 (5)	O-methylated flavonol O-hexoside (syringetin-type) (putative)	[12]
	N		507	329 (100), 344 (70), 345 (60)		
13	P	20.66	288	227 (100), 106 (50), 87 (20), 270 (10)	UI	(molecular ion not identified)

RT: retention time; *m*/*z*: mass-to-charge ratio; (**): confirmed by standards; (UI): unidentified compound.

### 2.2. Hemolytic Activity

The hemolytic activity of *M. rupestris* extracts was assessed at various concentrations (10, 5, 2.5, 1.25, and 0.625 mg/mL) and benchmarked against a positive hemolysis control (10% Triton X-100) and a negative control (2.5% Dimethyl sulfoxide DMSO). The negative control yielded 0% hemolysis rate (%HR), indicating the absence of activity, whereas the positive control resulted in 100% HR, thus confirming assay sensitivity as shown in Table 2.

### 2.3. Antitumoral Activity

The antitumoral activity of the berry extracts was evaluated using the MTT assay (3-[4,5-dimethylthiazol-2-yl]-2,5-diphenyl tetrazolium bromide) to assess cell proliferation. Cell proliferation of various tumor cell lines was measured after 72 h of exposure to *M. rupestris*, and dose–response curves were generated (Supplementary Figure S1). *M. rupestris* extract demonstrated inhibitory activity across all tested tumor cell lines. The calculated inhibitory concentration values (IC_50_) using dose–response curves (Table 3) revealed modest antiproliferative activity. *M. rupestris* exhibited IC_50_ values ranging from 10.4 to 22.5 mg/mL. Among these, cell lines derived from colorectal, hepatoma, and thyroid cancer (HCT116, HepG2, and THJ29T, respectively) showed the greatest sensitivity to the extract, having the lowest IC_50_ values. Moderate activity was observed against breast cancer (MDA-MB-231), while cervical cancer (HeLa) was the least responsive. Statistical analysis confirmed that the extract’s effect was primarily dose-dependent, with no significant differences between cell lines after multiplicity correction (Supplementary Table S1).

The IC_50_ values for tumor cells were similar across cell lines, resulting in low therapeutic index (TI) values, particularly in HeLa cells. The TI, calculated as the ratio of IC_50_ values between non-tumor and tumor cells, ranged from 0.8 to 1.8, with the highest selectivity observed in HepG2 cells. As a result, this extract exhibits a limited therapeutic window, which may not be sufficient to mitigate off-target toxicity.

### 2.4. Anti-Inflammatory Activity

**Figure 2:**
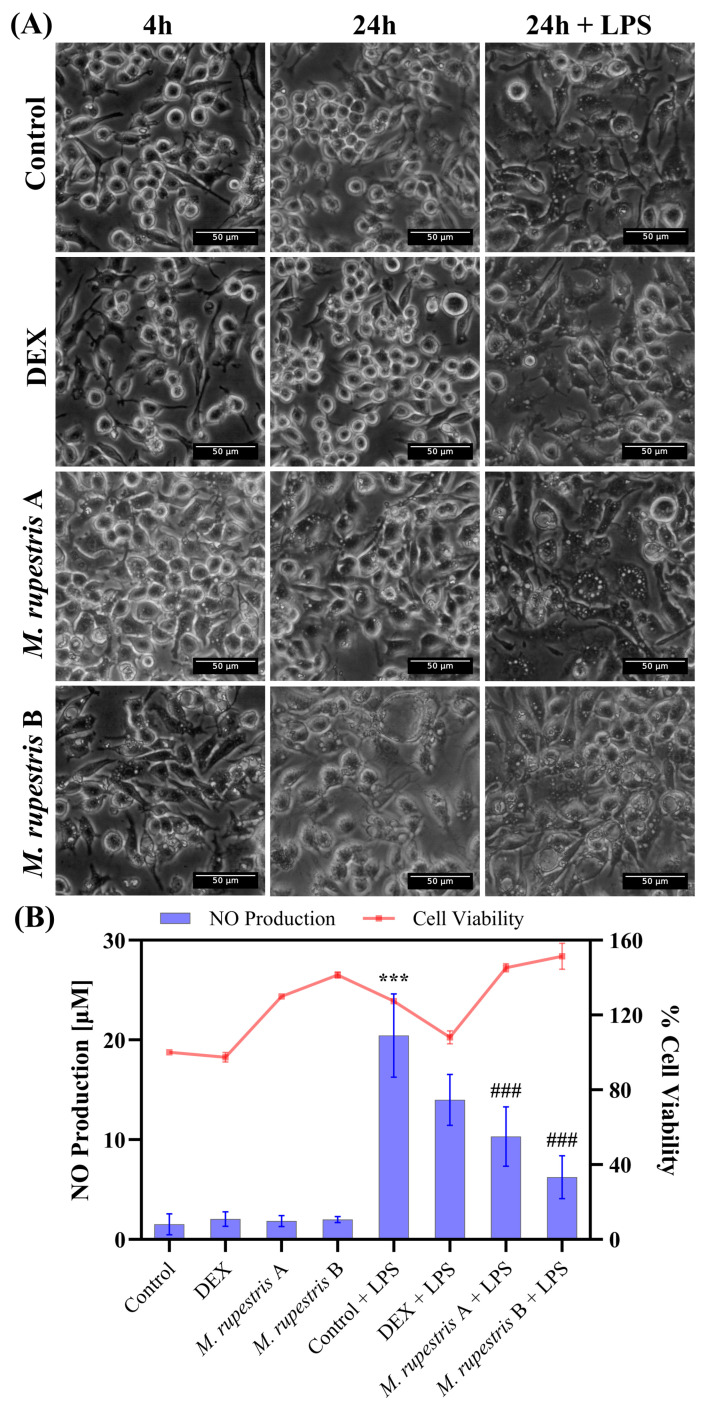
Effects of *M. rupestris* extract (A, 1 mg/mL; B, 2 mg/mL) on RAW 264.7 macrophages. (**A**) Morphological changes at 4 h and 24 h with or without LPS. (**B**) Nitric oxide production (left *y*-axis, bars) and cell viability (right *y*-axis, line) under the same conditions. Data are expressed as mean ± SD (n = 3). *** *p* < 0.001 compared with samples without LPS; ### *p* < 0.001 compared with samples stimulated with LPS.

To assess the effects of *M. rupestris*, RAW 264.7 cells were exposed for up to 24 h to two extract concentrations (A: 1 mg/mL; B: 2 mg/mL) with or without LPS. Phase-contrast microscopy (Figure 2A) showed early vacuolization at 4 h in extract-treated cells in both unstimulated and LPS-stimulated conditions; vacuolization intensified by 24 h. In DMEM and dexamethasone (DEX) controls, cell morphology remained unchanged at 4 h and 24 h, whereas the LPS group developed vacuolization by 24 h. Images at t = 0 h, which confirmed that cell morphology was indistinguishable from DMEM control at time 4 h, are provided in Supplementary Figure S2.

Regarding NO production (Figure 2B), the LPS control showed the highest NO induction (20.4 ± 4.1 µM). Treatment with *M. rupestris* extract reduced NO levels in LPS-stimulated cells in a concentration-dependent manner to 10.3 ± 2.9 µM at 1 mg/mL and 6.2 ± 2.1 µM at 2 mg/mL, corresponding to ~50% and ~70% reductions relative to LPS, respectively. DEX + LPS yielded 13.9 ± 2.5 µM (~32% reduction vs. LPS). Notably, this decrease in NO occurred alongside stress-associated morphological changes, regardless of LPS stimulation. In the absence of LPS, NO levels remained low (<2 µM), similar to DEX and DMEM alone (<2.5 µM). Cell viability results (Figure 2B) showed values above 97% for all conditions, with minor increases observed in *M. rupestris*-treated and LPS-stimulated cells. Statistical analysis revealed significant differences in NO production (*p* < 0.001) when comparing the LPS control (cells treated with LPS, maximal NO production) with untreated cells, as well as with *M. rupestris* extract under LPS stimulation (see Supplementary Information for statistical details).

### 2.5. Leishmanicidal and Cytotoxic Activity

The compound was evaluated against *Leishmania mexicana* promastigotes across a concentration range from 100 to 0.01 µg/mL (Table 4). Viability was expressed relative to the untreated RPMI control (100%). At 100 µg/mL, the compound did not reduce viability, which averaged 107.98% ± 9.70, suggesting a possible increase in metabolic activity. At 10, 1, and 0.1 µg/mL, viabilities were 82.56% ± 10.81, 81.02% ± 4.98, and 84.85% ± 8.50, respectively. The lowest concentration, 0.01 µg/mL, yielded 80.89% ± 5.34.

In contrast, amphotericin B markedly reduced viability to 12.83% ± 0.74, confirming the assay’s responsiveness. The vehicle control (2% DMSO) resulted in 103.38% ± 9.77 viability, indicating negligible cytotoxicity from the solvent. Coefficients of variation (CV) remained below 14% in all conditions, supporting the reproducibility of the results.

To assess potential host cell toxicity, RAW 264.7 macrophages were treated under the same concentration conditions. At the highest concentration (100 µg/mL), viability was 75.64% ± 5.08, while at 10, 1, and 0.1 µg/mL, values were 83.73% ± 3.37, 84.82% ± 5.49, and 87.28% ± 8.11, respectively. Interestingly, treatment at 0.01 µg/mL slightly increased viability to 101.62% ± 8.21.

Saponin (2.4 mg/mL) drastically reduced cell viability to 3.44% ± 0.14, confirming its cytotoxic potential. The DMEM control preserved viability at 100%, while the DMSO control showed 93.41% ± 5.49, indicating minimal solvent-associated toxicity. All CV values were under 10%, highlighting consistent experimental performance across replicates.

## 3. Discussion

The present study provides a comprehensive evaluation of the phytochemical composition and biological properties of *M. rupestris* extract. By integrating chemical profiling with in vitro assays, we aimed to establish correlations between the extract’s phenolic content and its functional effects.

The consistently low hemolytic activity of *M. rupestris* extracts across all concentrations indicates a minimal risk of red blood cell disruption (Table 2). This finding is particularly relevant for biomedical or cosmetic applications where contact with blood components is anticipated, reinforcing the biocompatibility of the extract [17]. These results support the hypothesis that *M. rupestris* extract, even at concentrations as high as 10 mg/mL, exert negligible hemolytic effects, with hemolysis levels consistently below 5%, classifying the extract as non-hemolytic. Such biocompatibility strengthens their candidacy for further pharmacological and biotechnological development [18].

The identification of several antioxidant metabolites in the extract provides a plausible biochemical basis for the low hemolytic activity observed. Quercetin is considered a potent antioxidant [19], and its derivatives, known to scavenge reactive oxygen species and protect lipid membranes from peroxidation, likely contribute to erythrocyte membrane stability. This antioxidant-mediated mechanism aligns with previous literature highlighting the protective role of such compounds in maintaining red blood cell integrity under oxidative stress conditions [20]. Regarding cellular antioxidant activity (CAA) assays, Markovinovi’c et al., 2024 examined the CAA of another member of the Ericaceae family, *Arbutus unedo* L. The ROS production was assessed in 4 cell types (HepG2, CAL 27, AGS, Caco-2) treated with the strawberry tree fruit extract (0.01–100 mg/mL) by using the 2′,7′-dichlorofluorescein-diacetate (DCFH-DA) assay and they demonstrated the capacity of the extract to inhibit ROS formation therefore they determined that *A. unedo* extract increases cellular survival and the antioxidant effect [21].

Likewise, anthocyanins such as malvidin-3-glucoside and petunidin-3-glucoside are known to integrate into lipid bilayers and enhance membrane robustness, potentially preventing hemolysis. Similarly, myricetin 3-O-hexoside has demonstrated inhibitory effects on lipid peroxidation, further substantiating its role in preventing cell lysis [22,23].

Taken together, the presence of these bioactive compounds suggests that the hemocompatibility of *M. rupestris* extract may not be merely a lack of cytotoxicity but an active process of membrane protection. This renders the extract particularly promising for applications involving oxidative stress, where preservation of erythrocyte function is paramount.

Research on the antitumor activity of the *Macleania* genus, particularly *M. rupestris*, is currently limited, with no documented evidence of its use in cancer treatment [6,24]. However, other berries within the *Ericaceae* family have been shown to exhibit antitumor activity, with numerous studies indicating that bioactive compounds found in edible berries from this family may reduce cancer risk, specifically in oral, colon, and liver cancers [3,25,26,27]. Although the IC_50_ values for *M. rupestris* indicate some antitumoral potential, its overall efficacy appears somewhat lower than similar berries (Table 3) [28,29,30], with consistently low TI across all tested tumor cell lines, likely influenced by its distinct phytochemical composition. Furthermore, the differential sensitivity observed among tumor cell lines may reflect intrinsic cellular differences that influence responsiveness to the extract, with selectivity arising from interactions between the bioactive compounds and tumor cell vulnerabilities determined by genotype, mitochondrial status, and apoptotic capacity [31,32,33,34]. Unlike other berries with robust antitumor properties driven by high levels of specific flavonoids and iridoids, and other phenolic compounds, these bioactive elements are present in *M. rupestris* but do not appear to significantly contribute to its antitumor activity [5,35,36]. Despite exhibiting strong antioxidant activity [4], the relatively low concentrations of anthocyanins in *M. rupestris*, which include delphinidin-3-arabinoside, petunidin-3-glucoside, cyanidin-3-glucoside, and malvidin-3-glucoside (Table 1), suggest that factors beyond antioxidant capacity influence its antitumoral efficacy. Similarly, flavonol glycosides such as myricetin-3-O-hexoside and methoxy-myricetin, along with quercetin glycosides like quercetin-3-galactoside and quercetin pentosides, have shown anticancer activity [37,38,39,40]. These compounds promote apoptosis and inhibit cancer cell proliferation, with methylation enhancing bioavailability and cytotoxicity, while glycosylation improves solubility and bioavailability, possibly contributing to their antitumor efficacy [41,42]. Yet, in *M. rupestris*, their apparent concentrations do not appear sufficient to induce meaningful cytotoxicity, although their presence may still support membrane protection, as seen in the hemolysis assay.

In addition to flavonoids, phenolic compounds like p-coumaric acid are noteworthy for their ability to induce apoptosis, inhibit cancer cell proliferation, suppress migration and invasion, exhibit anti-inflammatory effects, and potentially modulate epigenetic mechanisms, such as reactivating tumor suppressor genes or silencing oncogenes [43,44]. Coumaroyl iridoids and organic acids such as malic and tartaric acids, which are also present, may contribute modestly to the extract’s bioactivity, particularly through non-cytotoxic mechanisms such as pH modulation, disrupting cancer cell metabolism, and potentially enhancing the bioavailability of other active compounds in the extract [45,46,47]. However, their intrinsic antitumor action is modest, which could account for the observed low overall antitumor activity of the extract. Ultimately, the effectiveness of *M. rupestris* depends on factors such as the specific concentration, modifications, and interactions of the compounds in the extract [48]. Furthermore, the relatively lower sensitivity observed in cervical carcinoma cells may be influenced by differences in polyphenol uptake and by variations in the expression of molecular targets involved in apoptosis and proliferation [49,50].

The weak antitumor effects of *M. rupestris*, combined with its low TI, highlight its limited selectivity and potential cytotoxicity toward non-tumor cells. In contrast, its excellent hemocompatibility and antioxidant profile suggest greater value in applications prioritizing biocompatibility over direct anticancer action. This study represents the first report on the antitumoral activity of the extract from *M. rupestris*.

Nitric oxide is a key biomarker in inflammatory pathways, participating in essential processes such as tissue repair. Nevertheless, alterations in its normal production have been linked to chronic inflammatory disorders and cancer development [51,52]. The rich biodiversity of South America offers valuable opportunities to study fruits like *M. rupestris* and to evaluate their bioactivity in vitro. Identifying compounds with anti-inflammatory properties is particularly important for managing cases of physiological dysregulation [53]. RAW 264.7 cells exposed to two different concentrations of *M. rupestris* showed a dose–dependent reduction in NO levels, presenting a significant decrease compared to the dexamethasone control. Interestingly, the morphological changes followed the same dose–response trend, with increased vacuolization occurring with or without LPS stimulation. This effect could be attributed to quercetin derivatives, including quercetin-3-galactoside and quercetin-pentoside, identified in the extract in this and previous studies. These bioactive compounds have been reported to induce similar structural alterations in RAW 264.7 cells stimulated with LPS [54], attributed to F-actin cytoskeleton alterations without compromising cell viability. On the other hand, the vacuolization observed in LPS-stimulated RAW 264.7 macrophages in the absence of extracts constitutes a normal cellular response to innate immune activation, triggered by Toll-like receptor 4 (TLR4) [55]. This process involves the formation of autophagosomes and the de novo biosynthesis of sphingolipids [56,57], and is mechanistically linked to LPS-induced upregulation of inducible nitric oxide synthase (iNOS) and increased nitric oxide (NO) production, which can regulate autophagic and vesicular dynamics [58,59]. Together, these events potentiate intracellular degradation while maintaining cell viability [60].

Additionally, myricetin derivatives (myricetin-3-O-hexoside and methoxy-myricetin) present in *M. rupestris* may contribute to the observed anti-inflammatory activity, consistent with reports that pure myricetin suppresses nuclear factor kappa B (NF-κB) and mitogen-activated protein kinase (MAPK) signaling in RAW 264.7 macrophages and attenuates LPS-induced inflammation in vivo [61]. Anthocyanins, particularly cyanidin-3-glucoside, are also likely contributors, as they have been shown to decrease NO production, inhibit prostaglandin E2 (PGE2), reduce nitric oxide synthase (iNOS) and cyclooxygenase-2 (COX-2) expression, attenuate NF-κB activation, and increase inhibitor of kappa B alpha (I-κBα) levels in LPS-activated RAW 264.7 cells [62]. No information is currently available regarding the anti-inflammatory activity of *M. rupestris*. However, *Vaccinium floribundum*, which belongs to the same Ericaceae family and grows in South America, contains similar compounds, such as different forms of quercetin, myricetin, and cyanidin [63,64]. Nevertheless, studies have shown that *V. floribundum* does not significantly reduce NO production after LPS stimulation [65], nor does it affect the expression of iNOS or tumor necrosis factor α (TNF-α) after exposure to interferon gamma (IFN-γ) [65]. In contrast, *M. rupestris* significantly reduced NO production by approximately 50% at the lowest concentration tested, highlighting its potential anti-inflammatory effect under the specific experimental conditions employed in this study, although comparisons with other species should be made with caution. Interestingly, anthocyanin- and proanthocyanidin-enriched fractions from wild berries native to Alaska (*Ericaceae* family) have been reported to reduce intracellular ROS in LPS-stimulated RAW 264.7 macrophages [66], which may be relevant for further investigation in cell ROS response.

Macrophages RAW 264.7 were also used to evaluate cytotoxicity activity as a preliminary step for anti-leishmania potential evaluation. The extract induced moderate RAW 264.7 cytotoxicity at 100 µg/mL (75.64% ± 5.08 viability), while lower concentrations preserved or slightly enhanced cell viability. This biphasic behavior supports a hormetic model, in which low concentrations promote cell health while higher doses exert stress or mild toxicity. The *M. rupestris* extract showed no leishmanicidal activity against *Leishmania mexicana* promastigotes at concentrations ranging from 0.01 to 100 µg/mL. Interestingly, at the highest dose, viability increased to 107.98% ± 9.70, suggesting a potential metabolic stimulation rather than cytotoxicity. This response may be linked to antioxidant compounds present in the extract—such as quercetin, caffeic acid, and anthocyanins—which are known to influence mitochondrial function and redox homeostasis [67,68]. The differential effect between parasite and host cells may reflect differences in redox regulation or membrane susceptibility [69].

Overall, the lack of activity in promastigotes suggests that the extract’s antiparasitic potential may depend on host–parasite interactions not captured in axenic culture. The selective cytotoxicity toward macrophages and the presence of bioactive phytochemicals warrant further evaluation in intracellular amastigote models, where immunomodulatory or redox-mediated mechanisms could play a more prominent role [70,71].

### Limitations and Future Perspectives

This study provides an initial physicochemical and biological profile of the *M. rupestris* extract, underscoring its favorable safety characteristics and moderate anti-inflammatory activity while noting limited antiproliferative selectivity. In the present study, several molecular entities were identified as potentially associated with the observed biological activity. However, the precise chemical nature of the active compound(s) remains unresolved. To address this critical gap, future experimental efforts should focus on the fractionation of crude extracts followed by the systematic evaluation of individual purified components [72]. This approach will enable the elucidation of specific effects—either independent or synergistic—thereby allowing the unambiguous identification of the molecule(s) responsible for the observed biological phenomenon [72]. Such stratification is likely to improve potency (lower IC_50_), therapeutic index (TI), and allow future escalation into in vivo models [73].

Mechanistic work should couple targeted transcriptomics and proteomics with functional assays to define pathway engagement [74,75]. Rigorous in vivo validation can be essential to confirm efficacy, assess bioavailability, and establish longer-term safety. From a translational standpoint, the development of nutraceutical or phytopharmaceutical formulations (e.g., encapsulation to enhance stability and delivery) has impressive results, enhancing the potential of promising compounds [76,77]. Additionally, rational combination studies with approved agents could leverage the extract’s biocompatibility for chronic inflammatory or oxidative stress–related conditions [78,79]. Finally, coordinated work with Andean biodiversity programs may reveal chemotypic variants with superior yields or activity, accelerating both discovery and sustainable sourcing.

## 4. Materials and Methods

### 4.1. Plant Material and Physico-Chemical Analysis

*M. rupestris* (Kunth) A.C.Sm. berries (herbaria code: 4456, Herbario QUPS- Ecuador) were previously collected from the cloud forest Montano Alto, Cuenca-Ecuador (2°53′10.432″ S, 79°5′1.291″ O) (Project MAE-DNB-2019-0911-O), as reported in our previous study related to *M. rupestris* [4]. Briefly, the fruit was washed with plenty of water, and the dried berries were homogenized and divided into two groups. In the first group, weight, size, pH, soluble solids, titratable acidity, moisture, ash, and minerals of 20 berries were quantified. Ethereal extract (AOAC 922.06), calorific value (FAO), crude fiber (ICC113), total carbohydrate by difference (FAO), and protein (AOAC 2001.11) were also determined. The second portion was frozen at −80 °C and lyophilized in a Christ Alpha 1–4 LDplus (GmbH, Osterode am Harz, Germany). The dry powder was ground and stored until analysis.

### 4.2. Plant Extract

The extraction protocol used in this study was previously described by Barba-Ostria et al. [2] with some modifications. Briefly, the fruit was washed, gridded, and lyophilized to obtain powdered particles. For the ethanolic extraction, 10 g of the powdered material was combined with 200 mL of 96% ethanol. This mixture was then placed in an oil bath and agitated over a magnetic plate stirrer at 70 °C for 60 min.

Subsequently, the resulting solution was filtered using a solid–liquid filtration unit connected to a vacuum pump, and the filtrate was collected in a rotary evaporation flask. A total of 200 mL of 96% ethanol was added to the solid residue of the initial filtration and mixed until resuspension. This solution was filtered again, and the liquid filtrate was poured into the initial evaporation flask and mixed. The solution was evaporated in a rotary evaporator until the ethanolic solvent was eliminated. Finally, the remaining extract was lyophilized until complete desiccation, and the solid extract was weighted.

### 4.3. HPLC-MS/MS Analysis

The dry extract described previously was suspended in methanol-water (80:20), filtered using a 0.2 μm filter (RephiLe Bioscience Ltd., Acton, MA, USA), and used for HPLC-MS/MS analysis as previously described [80]. Briefly, the HPLC is a Vanquish (Thermo Fisher Scientific, Waltham, MA, USA) coupled to an LTQ-XL (Thermo Fisher Scientific, Waltham, MA, USA) and controlled by ￼Xcalibur software￼ (version 4.3). The stationary phase is an Accucore Vanquish C18 column (1.5 μm, 100 × 2.1 mm; Merck KGaA, Darmstadt, Germany) working at a constant temperature of 35 °C. The mobile phase consisted of a solution of 0.1% formic acid (A) and acetonitrile (B). The elution gradient was set as follows: 2% B, 0–4 min; 4% B, 4–22 min; 40% B, 22–32 min; 70% B, 32–40 min; 2% B, 40–45 min, and the column was re-balanced to the initial solvent conditions. The injection volume was 10 μL, and the flow rate was set to 0.2 mL/min. The mass range in DAD is recorded in the range from 50 to 2000 *m*/*z*. The electrospray ionization (ESI) conditions were capillary temperature of 275 °C, source voltage of 4.5 kV, capillary voltage of 18 V, and tube lens of 70. The sample was analyzed in positive and negative ionization modes. The tentative identification of the parent ions in positive ion mode was based on the fragmentation patterns. The patterns were compared with the MzCloud database￼￼ (https://www.mzcloud.org/ accessed on 5 Sep 2025), and those with a match score of more than 70% were selected. In addition, fragmentation patterns were identified by a literature search and the GNPS database (https://gnps.ucsd.edu/ accessed on 5 Sep 2025). Retention time was also considered for the tentative identification. Additionally, some commercial standards were also used, specifically quercetin (>95% HPLC, 551600, Sigma-Aldrich, St. Louis, MO, USA) and cyanidin 3-glucoside (R078F0, USP Reference Standard).

### 4.4. Hemolytic Activity Evaluation Assay

The hemolytic activity of the *M. rupestris* extract was assessed following a previously established protocol [81]. Briefly, ten milliliters of defibrinated sheep blood were subjected to three consecutive washes with PBS 1x. Following these washes, a 1% erythrocyte suspension in PBS 1x was prepared. This erythrocyte suspension was subsequently mixed in a 1:1 ratio with *M. rupestris* extract at the indicated concentrations, positive controls (10% Triton X-100), or negative controls (PBS 1x) in a 96-well polypropylene plate. The mixture was incubated at 37 °C for 1 h. Post incubation, the samples were centrifuged for 5 min at 1700× *g*. The supernatant was then carefully transferred to a transparent flat-bottom 96-well plate for absorbance measurement at 405 nm using a Cytation5 multi-mode plate reader (BioTek, Winooski, VT, USA). Each experiment included three technical replicates, and the entire procedure was repeated three times. For each sample, the hemolysis rate (HR%) was calculated according to the formula:

$$\mathrm{HR}\left(\%\right)\;=\;\frac{OD_{test}-OD_{neg}}{OD_{pos}-OD_{neg}}\times 100$$

The ASTM F756-00 criteria was used to clasify the hemolysis rate (HR%) as non-hemolytic (<5%), slightly hemolytic (5–10%), or hemolytic (>10%) [82].

### 4.5. Anti-Tumoral Activity Evaluation Assay

HeLa (human cervical carcinoma, ATCC No. CCL-2, RRID:CVCL_0030), HCT116 (human colorectal carcinoma, ATCC No. CCL-247, CVCL_0291), MDA-MB-231 (human breast adenocarcinoma, ATCC No. HTB-26, CVCL_0062), HepG2 (human hepatoma, ATCC No. HB-8065, RRID: CVCL_0027), and NIH3T3 (mouse NIH/Swiss embryo fibroblasts, ATCC No. CRL-1658, RRID: CVCL_0594) cell lines were obtained from the American Type Culture Collection (ATCC, Manassas, VA, USA). THJ29T (human thyroid carcinoma, Cat. No. T8254, RRID: CVCL_W922) was obtained from Applied Biological Materials Inc. (ABM, Richmond, BC, Canada). All cell lines were cultured in Dulbecco’s Modified Eagle Medium (DMEM/F12) (Corning, Corning in Manassas, VA, USA) supplemented with 10% fetal bovine serum (FBS) (Eurobio, Les Ulis, France) and 1% penicillin/streptomycin (Thermo Fisher Scientific, Gibco, Miami, FL, USA). All cell lines were maintained at 37 °C in a humidified atmosphere with 5% CO_2_.

To evaluate the impact of *M. rupestris* extract on cell proliferation, cells were seeded in 96-well plates at a density of 1 × 10^4^ cells/well and treated for 72 h with 100 μL of the extract at concentrations ranging from 0.1 to 20 mg/mL (dissolved in 100% DMSO, ensuring the final concentration did not affect cell viability), with each condition tested in quadruplicate. After the incubation period, cell viability was assessed using the MTT assay following standard protocols [83,84]. Briefly, 10 μL of MTT solution (5 mg/mL) was added to each well. After allowing 1–2 h of incubation at 37 °C in a humidified environment, the media was aspirated, and 50 μL of DMSO was added to each well to dissolve the formazan crystals. The mixture was gently agitated for 5 min before measuring the absorbance at 570 nm using a Cytation5 multi-mode detection system (BioTek, Winooski, VT, USA). Each data point was obtained from four replicates, expressed as mean ± standard deviation, and the experiment was replicated at least four times. To determine the concentration of the compound required to inhibit 50% of cell proliferation (IC_50_), dose–response curves were generated in GraphPad Prism 10.2 software (GraphPad Software, Inc., La Jolla, CA, USA). Additionally, the therapeutic index (TI) was calculated as the ratio between the IC_50_ of non-tumor cells (NIH3T3) and the IC_50_ of tumor cells.

### 4.6. Anti-Inflammatory Activity Evaluation Assay

RAW 264.7 cells (murine macrophages, ATCC No. TIB-71, RRID: CVCL 0493) were cultured in Dulbecco’s Modified Eagle Medium with 4.5 g/L glucose and L-glutamine (DMEM; Corning, Manassas, VA, USA) supplemented with 10% fetal bovine serum (FBS; Eurobio, Les Ulis, France) and 1% penicillin-streptomycin (Thermo Fisher Scientific, Gibco, Miami, FL, USA). For maintenance, a mixture of 30% conditioned medium and 70% fresh medium was used, as recommended by [85]. Cells were maintained at 37 °C in a humidified incubator with 5% CO_2_. For the anti-inflammatory assay, cells were seeded at 3 × 10^5^ cells/well in 24-well plates and allowed to adhere for 18–24 h, with two biological replicates for each condition. After this period, the medium was replaced with FBS-free DMEM containing the test compounds: DMEM alone as a control, dexamethasone (DEX; Sigma-Aldrich, St. Louis, MO, USA) at 2 μg/mL as an anti-inflammatory reference, and *M. rupestris* at two concentrations (A, 1 mg/mL; B, 2 mg/mL). These concentrations were selected based on the IC_50_ value determined for RAW 264.7 cells (1.47 mg/mL) via the MTT assay described in Section 4.5, with the corresponding dose–response curve provided in Supplementary Figure S3. One concentration was chosen below and one above the IC_50_ to evaluate effects within a physiologically relevant range. After 4 h, lipopolysaccharide (LPS; InvivoGen, San Diego, CA, USA) at 1 μg/mL was added to stimulate nitric oxide production. All stock solutions were prepared in DMEM. Cells were then incubated for an additional 20 h. Subsequently, 50 μL of the supernatant from each treatment was transferred to a 96-well plate, along with DMEM alone and a NO standard (Promega, Madison, WI, USA, G296A) from 0.78 μM to 100 μM. Then, 50 μL of Griess reagent (Sigma-Aldrich, G4410) at 50 mg/mL was added to each well, resulting in a final volume of 100 μL per well. After incubating for 10 min at room temperature in the dark, the absorbance was measured at 540 nm using a Cytation 5 plate reader (BioTek, Winooski, VT, USA). The DMEM alone absorbance was subtracted, and NO production was calculated based on the standard curve. Each experiment was performed in triplicate. Cell viability was assessed by fixing the 24-well plates with 4% paraformaldehyde for 20 min at room temperature before staining with 0.5% (*w*/*v*) crystal violet for 30 min at room temperature. Then, the plates were gently washed with water to eliminate the extra dye and left to dry at room temperature. Dried plates were scanned and quantified in a Cytation5 multimode detection system (BioTek, Winooski, VT, USA) at 570 nm. Untreated cells were used as a 100% cell viability control. Statistical analyses were performed using one-way ANOVA to evaluate the effect of treatments on NO production, conducted in Python version 3.15 with the Statsmodels library. Tukey’s HSD post hoc test was applied for multiple comparisons.

### 4.7. Leishmanicidal and Cytotoxic Activity Evaluation Assays

*Leishmania mexicana* promastigotes and RAW 264.7 murine macrophages were employed to evaluate the leishmanicidal and cytotoxic potential of the ethanolic crude extract following a protocol previously described. Promastigotes were cultured in RPMI-1640 medium supplemented with 10% fetal bovine serum (FBS) and 1% penicillin-streptomycin at 24 °C. RAW 264.7 cells were maintained in Dulbecco’s Modified Eagle Medium (DMEM) with 10% FBS and 1% penicillin-streptomycin at 37 °C in a humidified incubator with 5% CO_2_.

To assess compound activity, 1 × 10^6^ promastigotes in 100 μL of RPMI were seeded into V-bottom 96-well plates, and 6 × 10^3^ RAW 264.7 cells in 100 μL of DMEM were seeded into flat-bottom 96-well plates. After overnight incubation, cells were treated with 100 μL of the compound at final concentrations of 100, 10, 1, 0.1, and 0.01 μg/mL. Vehicle control consisted of 2% DMSO. Positive controls included amphotericin B (4 mg/mL) for parasites and saponin (2.4 mg/mL) for macrophages. Untreated wells with RPMI (for *Leishmania*) and DMEM (for RAW 264.7) served as negative controls. After 72 h of incubation under their respective conditions and protected from light, 20 μL of MTT solution (5 mg/mL in PBS) was added to each well, resulting in a final concentration of 0.45 mg/mL. Plates were gently agitated for 10 min, followed by incubation for 2 h under the same conditions. Then, samples were centrifuged at 4000 rpm for 10 min to precipitate formazan crystals. The supernatant was discarded, and 100 μL of DMSO was added to solubilize the formazan. Absorbance was measured at 570 and 630 nm using a microplate reader (BioTek, ELx808, Winooski, VT, USA). Each experiment was performed in technical triplicate and independently repeated in three biological replicates. Results were normalized against the corresponding untreated controls and expressed as a percentage of viability.

## 5. Conclusions

This study provides the first comprehensive phytochemical and biological characterization of *M. rupestris* ethanolic fruit extract. The chemical analysis revealed a diverse profile rich in anthocyanins, flavonols, and coumaroyl iridoids, which are compounds frequently associated with antioxidant and protective properties. In vitro assays demonstrated that the extract exhibits modest antitumoral activity with low therapeutic selectivity, negligible hemolytic activity, and a concentration-dependent reduction in nitric oxide production, highlighting its anti-inflammatory potential. Conversely, the extract did not display leishmanicidal activity under the tested conditions.

On the other hand, the results obtained regarding hemocompatibility and anti-inflammatory effects suggest that *M. rupestris* possesses good potential in these areas, which may support its potential applications in biomedical and nutraceutical contexts where safety and biocompatibility are essential. However, its relatively weak antiproliferative activity highlights the need for further research to optimize its efficacy, possibly through fractionation, enrichment of active metabolites, or evaluation of synergistic interactions.

## Figures and Tables

**Table 2:** Hemolytic activity of *M. rupestris* extract.

Extract Concentration (mg/mL)	%HR (Mean ± SD)
C-	0.0 ± 0.08
C+	100 ± 0.76
10	2.27 ± 0.21
5	2.23 ± 0.42
2.5	1.83 ± 0.80
1.25	1.73 ± 0.59
0.625	0.23 ± 0.2

%HR: hemolysis rate percentage; SD: standard deviation; C: control.

**Table 3:** Half maximal inhibitory concentration values (IC_50_) (mg/mL) against tumor and non-tumor cell lines at 72 h and therapeutic index (TI) values. Values are expressed as mean ± standard deviation, n = 4.

	HeLa	HCT116	MDAMB231	THJ29T	HepG2	NIH3T3
IC_50_	22.5 ± 0.7	11.1 ± 1.1	13.2 ± 0.5	11.7 ± 1.9	10.4 ± 1.3	11.1 ± 1.1
TI ^a^	0.8	1.7	1.4	1.6	1.8	-

^a^ IC_50_ (NIH3T3)/IC_50_ (tumor cell).

**Table 4:** Leishmanicidal and cytotoxic activity of *M. rupestris* extract.

Extract Concentration (µg/mL)	Viability (% ± SD)	Cell Type
Amphotericin B (inhibition control C+)	12.83 ± 0.74	*Leishmania mexicana* promastigote
DMSO 2%, (vehicle control, C−)	103.38 ± 9.77	
RPMI (untreated control, C−)	100	
100	107.98 ± 9.70	
10	82.56 ± 10.81	
1	81.02 ± 4.98	
0.1	84.85 ± 8.50	
0.01	80.89 ± 5.34	
Saponin (inhibition control, C+)	3.44 ± 0.14	RAW 264.7 macrophages
DMSO 2%, (vehicle control, C−)	93.41 ± 5.49	
DMEM (untreated control, C−)	100	
100	75.64 ± 5.08	
10	83.73 ± 3.37	
1	84.82 ± 5.49	
0.1	87.28 ± 8.11	
0.01	101.62 ± 8.21	

C+: positive control; C−: negative control; SD: standard deviation.

## Data Availability

The original data presented in the study are openly available in FigShare at DOI: 10.6084/m9.figshare.30107668.

## Supplementary Materials

The following supporting information can be downloaded at https://www.mdpi.com/article/10.3390/molecules30214251/s1, Figure S1. Dose-response curves of *M. rupestris* extract against tumor and non-tumor cell lines after 72 h incubation were generated using the results of the MTT assay, Figure S2. Phase-contrast microscopy of RAW 264.7 at time 0 h with all conditions used, Figure S3. Dose-response curves of *M. rupestris* extract against RAW 264.7 cell line after 72 h incubation were generated using the results of the MTT assay, Table S1. Statistical Analysis of Dose-Dependent Effects of M. rupestris Extract on Tumor and Non-Tumor Cell Viability. Table S2. NO production and cell viability were used for statistical analysis of anti-inflammatory activity.
