# Supplementary material for: Phytochemical Characterization and In Vitro Biological Activities of Macleania rupestris (Ericaceae): Insights into Nutraceutical Potential
Source: Molecules. 2025 Oct 31;30(21):4251. doi: 10.3390/molecules30214251 (PMC12608963; doi:10.3390/molecules30214251)
Supplement: Supplementary file 1 [file molecules-30-04251-s001.zip › molecules-3897863-supplementary.pdf]

molecules

Supplementary Material

Phytochemical Characterization and *In Vitro* Biological Activities of *Macleania rupestris* (Ericaceae): Insights into Nutraceutical Potential

Arianna Mayorga-Ramos [1], Rebeca Gonzalez-Pastor [1], Juan A. Puente-Pineda [1], Carlos Barba-Ostria [2,3], Eduardo Tejera [4,5], Diana Celi [4,5], Patricio Rojas-Silva [3], Ana Belén Peñaherrera-Pazmiño [1] and Linda P. Guamán [1]*

[1] Universidad UTE, Centro de Investigación Biomédica, Facultad de Ciencias de la Salud Eugenio Espejo, Quito, Ecuador; arianna.mayorga@ute.edu.ec (A.M.-R), rebeca.gonzalez@ute.edu.ec (R.G.-P), juan.puente@ute.edu.ec (J.A.P-P), ana.penaherrera@ute.edu.ec (A.B.P-P) and linda.guaman@ute.edu.ec (L.P.G).

[2] Escuela de Medicina, Colegio de Ciencias de la Salud, Universidad San Francisco de Quito, Quito, Ecuador; cbarbao@usfq.edu.ec (C.B-O) and projas1@usfq.edu.ec (P.R-S).

[3] Instituto de Microbiología, Colegio de Ciencias Biológicas y Ambientales COCIBA, Universidad San Francisco de Quito, Quito, Ecuador; cbarbao@usfq.edu.ec (C.B-O).

[4] Bio-Cheminformatics Research Group, Universidad de Las Américas, Quito, 170504, Ecuador; diana.celi@udla.edu.ec (D.C) and eduardo.tejera@udla.edu.ec (E.T.).

[5] Facultad de Ingeniería y Ciencias Aplicadas, Carrera de Biotecnología, Universidad de Las Américas, Quito, 170504, Ecuador; diana.celi@udla.edu.ec (D.C) and eduardo.tejera@udla.edu.ec (E.T.).

* Correspondence: linda.guaman@ute.edu.ec

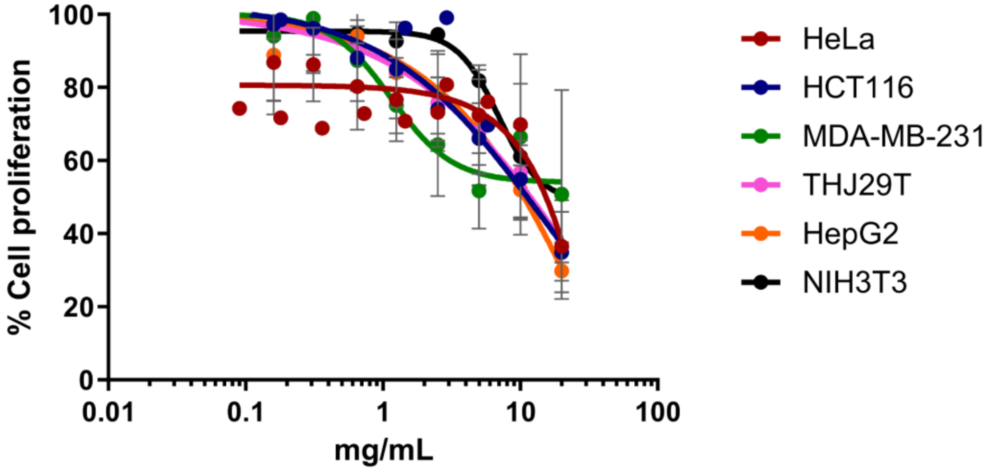

Supplementary Figure 1. Dose–response curves of *M. rupestris* extract against tumor and non-tumor cell lines after 72 h of incubation generated using the results of the MTT assay.

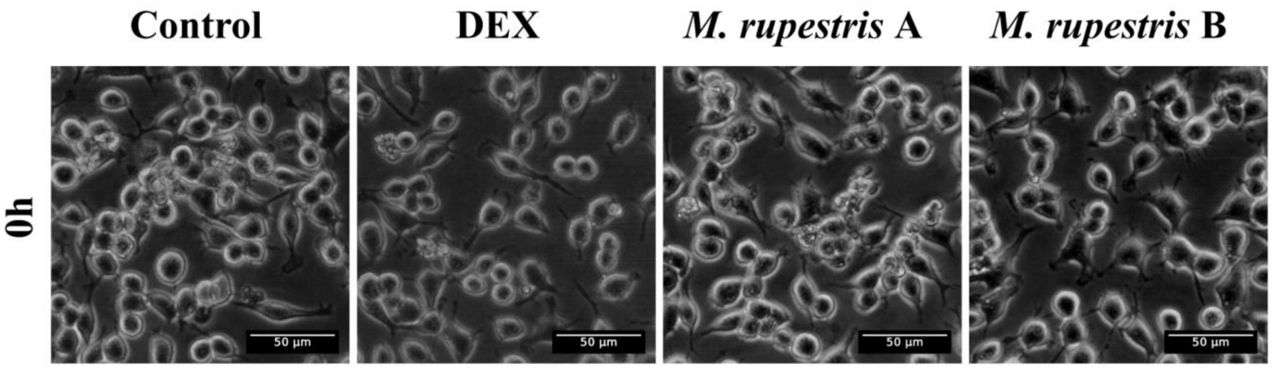

Supplementary Figure 2. Phase-contrast microscopy of RAW264.7 at time = 0 h with all conditions used.

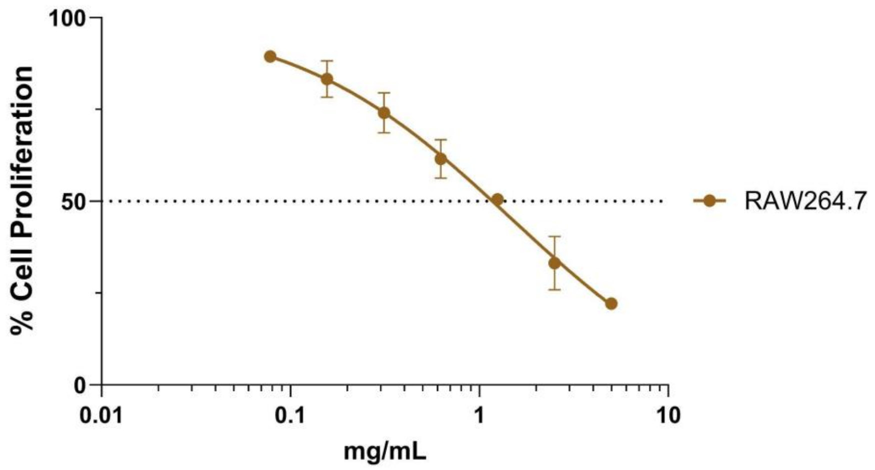

Supplementary Figure 3. Dose–response curves of *M. rupestris* extract against RAW 264.7 cell line after 72 h of incubation generated using the results of the MTT assay.

Supplementary Table 1. Statistical Analysis of Dose-Dependent Effects of *M. rupestris* Extract on Tumor and Non-Tumor Cell Viability.

	HeLa	HCT116	MDAMB231	THJ29T	HepG2	NIH3T3
**p-value (vs. NIH3T3)**	0.747	0.467	0.768	0.8662	0.8231	-
**Mean Diff (vs. NIH3T3)**	11.0931	-12.475	-10.7931	-9.1931	-9.9486	-

[a] Significant dose-dependent reduction in viability compared to control (0 mg/mL) at higher concentrations (Tukey's HSD, p-adj < 0.05 for doses ≥2.5 mg/mL; overall concentration effect: ANOVA $F(15,47)=16.71$, $p=5.60\times10^{-14}$). No significant pairwise differences between cell lines (Tukey's HSD, all p-adj > 0.05; cell line effect: ANOVA $F(5,47)=4.69$, $p=0.0015$).

Statistical Analysis of Dose-Dependent Effects of *M. rupestris* Extract on Tumor and Non-Tumor Cell Viability. A two-way ANOVA (factors: concentration, cell line) revealed a strong main effect of concentration ($F(15, 47) = 16.71$, $p = 5.60 \times 10^{-14}$) and a weaker but significant main effect of cell line ($F(5, 47) = 4.69$, $p = 0.0015$). Post hoc Tukey HSD comparisons relative to the untreated control (0 mg/mL) identified significant decreases in viability at 2.5 mg/mL (mean difference = −23.2, p_adj = 0.023), 5.0 mg/mL (−31.85, p_adj = 0.0002), 10.0 mg/mL (−39.8, p_adj < 0.001), and 20.0 mg/mL (−60.12, p_adj < 0.001). In contrast, no pairwise differences between individual cell lines reached significance after multiplicity correction (all p_adj > 0.05), indicating that the between-line effect size was small.

Supplementary Table 2. NO production and cell viability used for statistical analysis of anti-inflammatory activity.

**Conditions**	**NO Production (μM)**			**%Cell Viability**		
	Repetition 1	Repetition 2	Repetition 3	Repetition 1	Repetition 2	Repetition 3
**Control**	1.14	0.67	2.71	100	100	100
**DEX**	1.48	1.76	2.85	99.29577	98.40989	94.42623
***M. rupestris* A**	1.48	1.55	2.48	129.0493	129.1519	131.4754
***M. rupestris* B**	1.63	2.2	2.15	139.7887	142.9329	141.4754
**Control + LPS**	16.21	20.53	24.58	126.5845	128.0919	127.7049

**DEX + LPS**	12.19	15.8	-	104.4014	111.3074	108.3607
***M. rupestris* A + LPS**	7.51	9.95	13.45	143.662	144.3463	147.7049
***M. rupestris* B + LPS**	4	6.38	8.31	148.0634	146.8198	159.3443

A one-way ANOVA revealed a robust effect of treatment on NO production ($F(7,15) = 29.24$, $p = 1.21 \times 10^{-7}$; SS_between = 994.09; SS_within = 72.86). Tukey's HSD post hoc analysis showed significant reductions compared with the LPS control for extract A + LPS (mean difference = −10.14 µM, p_adj = 0.0009; 95% CI −16.42 to −3.85) and extract B + LPS (−14.21 µM, p_adj < 0.001; 95% CI −20.50 to −7.92). The reduction observed with DEX + LPS did not remain significant after multiplicity correction (−6.45 µM, p_adj = 0.084). LPS stimulation significantly increased NO relative to untreated control (+18.93 µM, p_adj < 0.001). Extracts applied without LPS did not differ from control (e.g., extract A alone vs. control: +0.33 µM, p_adj = 1.0). The contrast between LPS-stimulated extracts (A + LPS vs. B + LPS) was not significant (−4.07 µM, p_adj = 0.371).
